# Leukocyte elastase, a1-proteinase inhibitor, and autoantibodies to neuroantigens in diagnostics of endogenous depressive disorders

**DOI:** 10.1192/j.eurpsy.2022.1804

**Published:** 2022-09-01

**Authors:** Z. Sarmanova, N. Subbotskaya, S. Zozulya, A. Barkhatova, T. Klyushnik

**Affiliations:** 1 Mental Health Research Centre, Laboratory Of Neuroimmunology, Moscow, Russian Federation; 2 Mental Health Research Centre, Department Of Endogenous Mental Disorders And Affective Conditions, Moscow, Russian Federation

**Keywords:** endogenous depressive disorders, leukocyte elastase, a1-proteinase inhibitor, autoimmune reactions

## Abstract

**Introduction:**

It has been suggested that the activation of systemic inflammatory response in depression is associated with inflammatory changes in the brain (neuroinflammation) and may reflect the severity of the clinical symptoms in patients.

**Objectives:**

To study the relationship between clinical and immune parameters in patients with endogenous depressive disorders for the possible use of these indicators for diagnostics of these conditions.

**Methods:**

Patients with bipolar affective disorder (group 1) and recurrent depressive disorder (group 2) (F31, F32, F33) were examined before the therapy. Mentally healthy age- and gender-matched persons were investigated as controls. The severity of depressive symptoms was assessed by HDRS. The activity of inflammatory indicators (leukocyte elastase (LE) and 1-proteinase inhibitor (ɑ1-PI)), as well as the level of autoantibodies (AB) to S-100B and MBP, were measured in plasma.

**Results:**

Group 1 was characterized by an increase of LE and ɑ1-PI activity in comparison with the control group (р<0.001; р=0.002) and group 2 (р<0.05). No significant difference in AB to neuroantigens was found. Group 2 was distinguished by the increase in activity of the inflammatory indicators (р<0.01; р<0.05) as well as the autoimmune reactions to neuroantigens compared with control one (р=0.03). The correlations between complex assessment of the immune system and the severity of depressive symptoms in both groups were revealed (χ2=6.1; p=0.013; χ2=4.8; p=0.05).

**Conclusions:**

Revealed correlations suggest that inflammatory markers are involved in the pathogenesis of endogenous depressive disorders and can be used as an additional differential diagnostics criterion for the assessment of the clinical state of patients.

**Disclosure:**

No significant relationships.

